# Significant Benefits of *AIP* Testing and Clinical Screening in Familial Isolated and Young-onset Pituitary Tumors

**DOI:** 10.1210/clinem/dgaa040

**Published:** 2020-01-30

**Authors:** Pedro Marques, Francisca Caimari, Laura C Hernández-Ramírez, David Collier, Donato Iacovazzo, Amy Ronaldson, Kesson Magid, Chung Thong Lim, Karen Stals, Sian Ellard, Ashley B Grossman, Márta Korbonits, FIPA Consortium, Prakash Abraham, Elena Aflorei, Amar Agha, James Ahlquist, Scott A Akker, Krystallenia Alexandraki, Sándor Alföldi, João Anselmo, Wiebke Arlt, Brew Atkinson, Anna Aulinas-Masó, Simon J Aylwin, Atik Baborie, Philippe F Backeljauw, Corin Badiu, Stephanie Baldeweg, Steve Ball, Gul Bano, Ariel Barkan, John Barton, Julian Barwell, Peter Bates, Carmen Bernal-González, Michael Besser, John S Bevan, Alex Bickerton, Jo Blair, Marek Bolanowski, Pierre Bouloux, Lisa Bradley, Karin Bradley, Caroline Brain, Antonia Brooke, Roger Brown, Michael Buchfelder, Christine Burren, Mehtap Cakir, Natalie Canham, Joel Capraro, Paul Carroll, Philippa Carter, David Carty, Dominic Cavlan, Harvinder S Chahal, Tim Cheetham, Farida Chentli, Catherine Choong, Mirjam Christ-Crain, Teng-Teng Chung, Peter Clayton, Richard N Clayton, Mark Cohen, Hamish Courtney, David Cove, Elizabeth Crowne, Daniel Cuthbertson, Jacob Dal, Nadezhda Dalantaeva, Svetozar Damjanovic, Christina Daousi, Ken Darzy, Mehul Dattani, Michaela Davies, Justin Davies, Julian Davis, Margaret de Castro, Laura de Marinis, Cheri Deal, Judit Dénes, Paul Dimitri, Neil Dorward, Graham Dow, William Drake, Maralyn Druce, Juliana Drummond, Pinaki Dutta, Larisa Dzeranova, Britt Edén-Engström, Rosalind Eeles, Maria Elfving, Kate Ellis, Marianne Elston, Louise Emmerson, Shereen Ezzat, Naomi Fersht, Simona Fica, Stefan Fischli, Maria Fleseriu, Elizabeth Forsythe, William Foulkes, Pamela Freda, Theodore Friedman, Mónica Gadelha, Mary Gainsborough, Stephen Gallacher, Patricia Gallego, Hoong-Wei Gan, Carmen Georgescu, Evelien Gevers, Catherine Gilkes, Nigel Glynn, James E Goldman, Anthony P Goldstone, Miklós Góth, Andrew Green, Lynn Greenhalgh, Joan Grieve, Luiz Griz, Mirtha Guitelman, Alper Gürlek, Mark Gurnell, Peter Shane Hamblin, Vaclav Hana, Philip Harding, Eleanor Hay, David A Hilton, Winnie Ho, Greg Hong, Katalin Horváth, Simon Howell, Trevor A Howlett, Charlotte Höybye, Steven Hunter, Chandi Idampitiya, Péter Igaz, Ali Imran, Warrick J Inder, Takeo Iwata, Louise Izatt, Sujatha Jagadeesh, Colin Johnston, Biju Jose, Gregory Kaltsas, Felicity Kaplan, Niki Karavitaki, Darko Kastelan, Michelle Katz, Tara Kearney, Melanie Kershaw, Bernard Khoo, Cathy Kiraly-Borri, Robertas Knispelis, Gábor L Kovács, Anand Kumar, Ajith V Kumar, Imre Zoltan Kun, Angelos Kyriaku, Ioana Lambrescu, Anne Katrin Lampe, Edward R Laws, Agnieszka Lebek-Szatanska, Ronald M Lechan, Graham Leese, Andrew Levy, Miles J Levy, Krzysztof Lewandowski, Eleanor Lin, Janet Lo, Catherine Lyons, Niki Maartens, Mohamad Maghnie, Taffy Makaya, Hani Marcus, Marek Niedziela, Niamh Martin, Akira Matsuno, Barbara McGowan, Siobhán E McQuaid, Milica Medic-Stojanoska, Nigel Mendoza, Moisés Mercado-Atri, Sachith Mettananda, Emese Mezősi, Dragana Miljic, Karen K Miller, Silvia Modenesi, Mark E Molitch, John Monson, Damian G Morris, Patrick J Morrison, Barbara Mosterman, Alia Munir, Robert D Murray, Madalina Musat, Nina Musolino, Lisa Nachtigall, Dinesh Nagi, Ramesh Nair, Richard Nelson, John Newell-Price, Khash Nikookam, Arla Ogilivie, Steve M Orme, Martin O´Weickert, Aparna Pal, Ionela Pascanu, Attila Patócs, Catherine Patterson, Simon H Pearce, Francesca Pecori Giraldi, Lynette Penney, Luis Gustavo Perez-Rivas, Marija Pfeifer, Fraser Pirie, Nicola Poplawski, Vera Popovic, Michael Powell, Peter Pullan, Richard Quinton, Serban Radian, Harpal Randeva, Narendra Reddy, Aled Rees, Valerie Renals, António Ribeiro de Oliveira, Tristan Richardson, Celia Rodd, Richard J M Ross, Federico Roncaroli, Fiona Ryan, Roberto Salvatori, Christof Schöfl, Debbie Shears, Kevin Shotliff, Robert Skelly, Katie Snape, Beatriz S Soares, Noel Somasundaram, Anna Spada, James Sperber, Helen Spoudeas, Maria Stelmachowska-Banas, Susan Stewart, Helen L Storr, Christian Strasburger, Maria Elisabeth Street, Isabelle Suter-Widmer, Graeme Suthers, Francesca Swords, Luis V Syro, Brede Swantje, Candy Sze, Juliet Taylor, Rajesh V Thakker, Elaine Tham, Chris Thompson, Michael O Thorner, Miklós Tóth, Peter J Trainer, Stylianos Tsagarakis, Gina Twine, Marinella Tzanela, Janos Vadasz, Bijay Vaidya, Vladimir Vaks, Mary Lee Vance, Rasa Verkauskiene, Hilde Von Esch, John A Wass, Mona Waterhouse, Susan Webb, Astrid Weber, Florian Wernig, Hakan Widell, Shozo Yamada, Patrick Yap, Sema Yarman, Philip Yeoh, Katsuhiko Yoshimoto, Kevin Yuen, Nicola N Zammitt

**Affiliations:** 1 Centre for Endocrinology, William Harvey Research Institute, Barts and the London School of Medicine and Dentistry, Queen Mary University of London, London, UK; 2 Section on Endocrinology & Genetics, Eunice Kennedy Shriver National Institute of Child Health and Human Development (NICHD), National Institutes of Health (NIH), Bethesda, Maryland; 3 Exeter Genomics Laboratory, Royal Devon & Exeter NHS Foundation Trust, UK

**Keywords:** pituitary neuroendocrine tumor, pituitary adenoma, familial isolated pituitary adenoma, somatotropinoma, gigantism, aryl hydrocarbon receptor-interacting protein

## Abstract

**Context:**

Germline mutations in the aryl hydrocarbon receptor-interacting protein (*AIP*) gene are responsible for a subset of familial isolated pituitary adenoma (FIPA) cases and sporadic pituitary neuroendocrine tumors (PitNETs).

**Objective:**

To compare prospectively diagnosed *AIP* mutation-positive (*AIP*mut) PitNET patients with clinically presenting patients and to compare the clinical characteristics of *AIP*mut and *AIP*neg PitNET patients.

**Design:**

12-year prospective, observational study.

**Participants & Setting:**

We studied probands and family members of FIPA kindreds and sporadic patients with disease onset ≤18 years or macroadenomas with onset ≤30 years (n = 1477). This was a collaborative study conducted at referral centers for pituitary diseases.

**Interventions & Outcome:**

*AIP* testing and clinical screening for pituitary disease. Comparison of characteristics of prospectively diagnosed (n = 22) vs clinically presenting *AIP*mut PitNET patients (n = 145), and *AIP*mut (n = 167) vs *AIP*neg PitNET patients (n = 1310).

**Results:**

Prospectively diagnosed *AIP*mut PitNET patients had smaller lesions with less suprasellar extension or cavernous sinus invasion and required fewer treatments with fewer operations and no radiotherapy compared with clinically presenting cases; there were fewer cases with active disease and hypopituitarism at last follow-up. When comparing *AIP*mut and *AIP*neg cases, *AIP*mut patients were more often males, younger, more often had GH excess, pituitary apoplexy, suprasellar extension, and more patients required multimodal therapy, including radiotherapy. *AIP*mut patients (n = 136) with GH excess were taller than *AIP*neg counterparts (n = 650).

**Conclusions:**

Prospectively diagnosed *AIP*mut patients show better outcomes than clinically presenting cases, demonstrating the benefits of genetic and clinical screening. *AIP*-related pituitary disease has a wide spectrum ranging from aggressively growing lesions to stable or indolent disease course.

Pituitary neuroendocrine tumors (PitNETs) are relatively common (~1:1000 clinically relevant cases in the general population) and familial cases represent around 5% of this patient cohort (1,2). Familial isolated pituitary adenoma (FIPA) is a heterogeneous condition that involves the presence of PitNETs in 2 or more members of the same family without other syndromic manifestations. Up to 20% of all FIPA and 50% of familial acromegaly kindreds carry germline mutations in the aryl hydrocarbon receptor-interacting protein (*AIP*) gene (1,3,4). These mutations are also seen in sporadically diagnosed PitNETs (simplex cases), particularly in young patients, where the lack of family history is usually due to incomplete penetrance rather than de novo mutations (5–8). The typical *AIP* mutation-positive (*AIP*mut) phenotype is characterized by a young patient presenting with a large invasive growth hormone (GH)-secreting PitNET that is refractory to conventional treatments (1,3–5,9–11), with *AIP*mut somatotropinomas being responsible for 29% of pituitary gigantism cases (12).

Family members at risk of inheriting an *AIP* mutation are recommended to undergo genetic testing and carriers are referred for clinical screening of pituitary disease (1,3,13–15). The rationale behind this strategy is that identifying PitNETs in *AIP*mut carriers with otherwise unrecognized disease at an early stage increases the likelihood of effective treatment and remission (1,3,14). The assumption is that screening-discovered PitNETs (ie, prospectively diagnosed PitNETs) are diagnosed at a less advanced stage and are less invasive than PitNETs with a clinical presentation, and thus should show a more favorable response to treatment and better clinical outcomes. However, these predicted advantages have never been actually shown in a prospective study.

Here, we present the results of a 12-year follow-up study on a large cohort of *AIP*mut patients, where we have characterized prospectively diagnosed *AIP*mut PitNET patients compared with clinically presenting cases. Our results highlight the critical importance of *AIP*mut genetic screening in selected individuals, and of clinical follow-up in known *AIP*mut carriers. Furthermore, we have expanded the description of *AIP*mut PitNET phenotype, disease course, and outcomes compared with *AIP*neg cases.

## Materials and Methods

### Study population

We selected our study population from our cohort (2079 patients with PitNETs and their 1029 unaffected relatives) recruited via the collaborative research network of the International FIPA Consortium (collaborators listed at the end of the manuscript) between February 2007 and April 2019; details of recruitment have been previously described (3). All participants gave written informed consent approved by the local ethics committee. Indications for *AIP* genetic testing were (1) patients with FIPA; (2) macroadenomas with disease onset at ≤30 years; and (3) PitNETs with disease onset at ≤18 years. First-degree family members of individuals carrying *AIP* mutations were offered genetic testing. We included in our analysis all patients with known *AIP* mutational status matching these criteria (n = 1477). We excluded patients with undetermined affected status (ie, proven *AIP*mut carriers who did not undergo clinical screening or had pending clinical test results by the time of data analysis). Patients with X-linked acrogigantism or syndromic disease (multiple endocrine neoplasia type 1 (MEN1), MEN4, Carney complex, SDHx-related, McCune–Albright and DICER1 syndromes, identified on the basis of clinical, biochemical, and genetic testing as appropriate) were excluded.

Of 1477 patients included in the study, 167 were *AIP*mut (33 not reported previously (11)), 154 with documented germline *AIP* pathogenic/likely pathogenic variant, and 13 affected subjects with predicted *AIP*mut status (obligate carriers in *AIP*mut kindreds but not formally tested, including subjects already deceased). Pathogenicity of *AIP* variants was determined as previously described (3,11); only pathogenic or likely pathogenic variants were considered to be “mutations”. The *AIP*neg subgroup included 1310 patients with PitNETs in whom a germline *AIP* mutation was excluded by genetic testing of all simplex probands and of the youngest affected member in the families.

### Genetic testing and clinical screening

*AIP* testing was performed using either Sanger sequencing and multiplex ligation-dependent probe amplification, or targeted next-generation sequencing on genomic DNA obtained from blood or saliva samples (3,11,16). All the unaffected individuals with positive genetic screening for *AIP* were advised to undergo clinical, biochemical, and image screening tests by their local physician for the early diagnosis of possible pituitary disease. Follow-up was advised on an annual basis or as appropriate (1,3,13).

### Study groups and clinical parameters

The familial cohort comprised patients with FIPA. The sporadic cohort included patients with young onset PitNETs (≤30 years) with no known family history of PitNETs or syndromic disease. The clinical diagnoses were established as GH excess (acromegaly and gigantism), prolactinomas, clinically nonfunctioning (NF)-PitNETs, Cushing’s disease, and thyrotropinomas, as previously described (3). Cases where the diagnosis was not specified due to unavailability of histopathological, clinical, or biochemical data were termed “PitNET not specified (NS)”. Age of onset was defined as the age of presentation of first symptoms. Macroadenomas were defined as tumor size ≥10 mm. Hypopituitarism at diagnosis and at last follow-up was defined as the presence of at least 1 pituitary deficiency documented biochemically. The number of treatments included the number of individual treatments received (each medication, surgery, and radiotherapy). Multimodal treatment was defined as the employment of 2 or more distinct forms of treatment in patient management. The reoperation subgroup involved patients who had at least 1 additional surgery following their first operation. Active disease was considered in patients with secretory PitNETs displaying the respective pituitary hormone above the normal assay range, and/or evidence of persistent or recurrent progressive tumor remnants in the surveillance pituitary magnetic resonance imaging (MRI) scans for both secretory PitNETs and NF-PitNETs. Small persistent tumor remnants after operation, stable over a period of time, and requiring no further intervention were considered as not active NF-PitNETs.

### Statistical analysis

Qualitative variables were expressed as percentages and analyzed with the χ ^2^ test to compare 2 or more groups. Quantitative or continuous variables were tested for Gaussian distribution with the Shapiro–Wilk test, and nonparametric and parametric data were then further analyzed with the Mann–Whitney U and Student t-tests, respectively. *P *< .05 was considered statistically significant. Statistical analyses were carried out using the SPSS software version 20 (IBM, USA) and GraphPad version 6 (Prism, USA). Data are presented as mean and standard deviation for continuous variables and as percentages for categorical variables.

## Results

### General characterization of the study population

**Figure 1.**
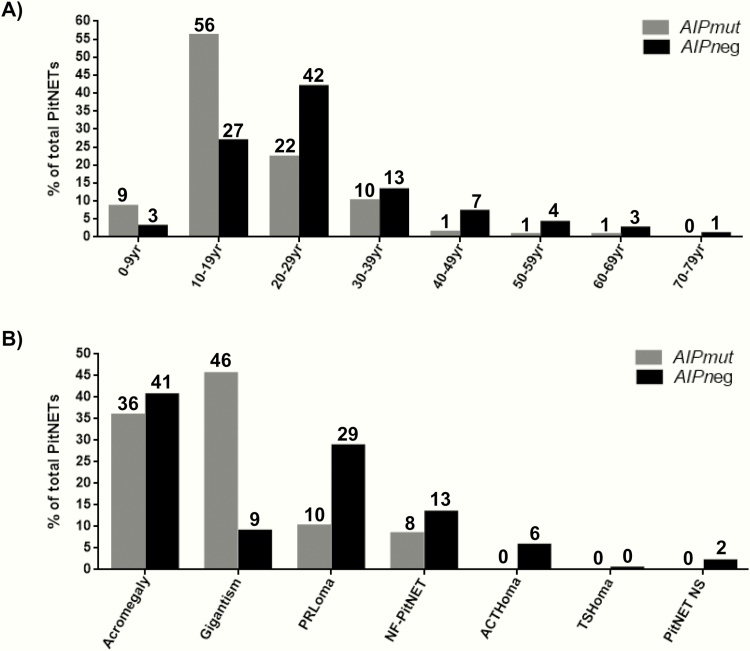
Distribution of *AIP*mut vs *AIP*neg PitNETs according to age at onset (A) and to clinical diagnosis (B). Numbers above columns represent percentage of patients. We note that the two *AIP*mut cases with first symptoms in the 5th and 6th decade, both had macroprolactinomas, 1 presenting with apoplexy. ACTHoma, ACTH-secreting adenoma or Cushing’s disease; *AIP*mut, *AIP* mutation-positive; *AIP*neg, *AIP* mutation-negative; PitNET NS, pituitary neuroendocrine tumor not specified; NF-PitNET, non-functioning PitNET; PRLoma, prolactinoma; TSHoma, thyrotropinoma; yr, years.

Of the 1477 patients with PitNETs, 167 were *AIP*mut (11.3%), and 1310 were *AIP*neg patients (FIPA or age ≤30 years at onset). Demographic and clinical characteristics and comparative analysis of *AIP*mut vs *AIP*neg PitNETs are presented in Table 1 and Fig. 1. The familial cohort (355 families, 700 patients, 47% of the whole study population) consisted of 37 *AIP*mut kindreds (114 patients) and 318 *AIP*neg families (586 patients). Of the 37 *AIP*mut families, 36 (97.8%) had at least 1 somatotropinoma case, 19 were homogeneous somatotropinoma kindreds, and 1 was homogeneous prolactinoma family. Of the 318 *AIP*neg families, 146 (46%) were homogeneous and 172 were heterogeneous, with detailed subtypes shown in Table 2. In the sporadic cohort (n = 777), 53 (6.8%) had an *AIP* mutation (Table 1). Within the sporadic tumor subgroup, 10.5% (50 out of 477) of somatotropinomas, 1.5% (3 out of 197) of prolactinomas, and none (0 out of 54) of the NF-PitNET cases were found to harbor a germline *AIP* mutation (Table 3; all supplementary material and figures are located in a digital research materials repository (17)).

**Table 1.** Characteristics of the study population and comparative analysis of *AIP*mut vs *AIP*neg patients

	*AIP*mut vs *AIP*neg PitNETs			
	*AIP*mut	*AIP*neg		
	n = 167	n = 1310	*P*	Overall study population n = 1477
Cohort type based on family history of PitNETs				
Familial cohort	68.3	44.7	**<.001**	47.4
Sporadic cohort	31.7	55.3		52.6
Gender				
Male	61.1	45.2	**<.001**	47.0
Female	38.9	54.8		53.0
Age at disease onset ≤18 yr	64.8	28.8	**<.001**	33.1
Age at first symptoms (yr)	19.0 ± 9.5	26.8 ± 13.1	**<.001**	25.9 ± 13.0
Age at diagnosis (yr)	24.3 ± 11.9	30.0 ± 13.5	**<.001**	29.4 ± 13.5
Delay in diagnosis (yr)	4.1 ± 6.6	3.2 ± 4.9	.212	3.3 ± 5.1
GH excess	81.4	49.6	**<.001**	53.2
Pituitary apoplexy	8.2	3.6	**.009**	4.2
Hypopituitarism at diagnosis	42.7	49.0	.318	47.9
Number of pituitary deficiencies at diagnosis	0.84 ± 1.11	0.79 ± 1.03	.841	0.80 ± 1.05
Macroadenoma	83.2	79.2	.259	79.7
Maximum tumor diameter (mm)	20.1 ± 13.0	22.8 ± 16.0	.281	22.5 ± 15.7
Suprasellar extension	54.3	42.4	**.043**	43.9
Cavernous sinus invasion	36.7	28.3	.122	29.3
Ki-67 > 3%	41.4	41.0	.972	41.1
Number of treatments	2.07 ± 1.66	1.87 ± 1.32	.228	1.90 ± 1.38
Number of surgeries	0.93 ± 0.79	0.87 ± 0.72	.468	0.88 ± 0.73
Reoperation	23.1	16.9	.106	17.8
Radiotherapy	32.9	21.5	**.002**	23.2
Multimodal treatment	67.2	47.0	**<.001**	49.7
≥3 treatments	40.3	25.8	**<.001**	27.7
Active disease at last follow-up	25.0	34.5	**.041**	32.8
Hypopituitarism at last follow-up	29.6	33.6	.574	32.9
Number of pituitary deficiencies at last follow-up	0.45 ± 0.96	0.77 ± 1.27	.148	0.71 ± 1.22
Follow-up duration (yr)	11.2 ± 12.3	7.8 ± 9.5	**.008**	8.4 ± 10.1

Categorical data are shown as %; continuous variables are shown as mean ± standard deviation. *P* values in bold are those < .05 (statistically significant).

Abbreviations: *AIP*mut, *AIP* mutation-positive; *AIP*neg, *AIP* mutation-negative; GH, growth hormone; PitNET, pituitary neuroendocrine tumor; yr, years.

**Table 2.** *AIP*mut and *AIP*neg FIPA kindreds according to pituitary tumor types

	*AIP*mut kindreds	*AIP*neg kindreds	Total
PitNET types within the same kindred	n = 37	n = 318	n = 355
ACTHoma only	0	7 (2.2)	7 (2.0)
ACTHoma + FSHoma	0	1 (0.3)	1 (0.3)
ACTHoma + GHoma	0	7 (2.2)	7 (2.0)
ACTHoma + NF-PitNET	0	1 (0.3)	1 (0.3)
ACTHoma + NF-PitNET + PitNET NS	0	1 (0.3)	1 (0.3)
ACTHoma + NF-PitNET + PRLoma	0	1 (0.3)	1 (0.3)
ACTHoma + PitNET NS	0	2 (0.6)	2 (0.6)
ACTHoma + PRLoma	0	8 (2.5)	8 (2.3)
GHoma only	19 (51.4)	68 (21.4)	87 (24.5)
GHoma + NF-PitNET	8 (21.6)	25 (7.9)	33 (9.3)
GHoma + NF-PitNET + PRLoma	1 (2.7)	4 (1.3)	5 (1.4)
GHoma + PitNET NS	0	19 (6.0)	19 (5.3)
GHoma + PitNET NS + PRLoma	0	1 (0.3)	1 (0.3)
GHoma + PRLoma	8 (21.6)	45 (14.2)	53 (14.9)
NF-PitNET only	0	24 (7.5)	24 (6.8)
NF-PitNET + PitNET NS	0	14 (4.4)	14 (3.9)
NF-PitNET + PRLoma	0	24 (7.5)	24 (6.8)
PRLoma only	1 (2.7)	47 (14.8)	48 (13.5)
PRLoma + FSHoma	0	1 (0.3)	1 (0.3)
PRLoma + PitNET NS	0	18 (5.7)	18 (5.1)

Data are shown as n (%).

Abbreviations: ACTHoma, ACTH-secreting adenoma or Cushing’s disease; *AIP*mut, *AIP* mutation-positive; *AIP*neg, *AIP* mutation-negative; FSHoma, FSH-secreting adenoma; GHoma, GH-secreting adenoma or somatotropinoma (this category includes acromegaly and gigantism cases); PitNET NS, pituitary neuroendocrine tumor not specified; NF-PitNET, nonfunctioning PitNET; PRLoma, prolactinoma.

**Table 3.** Comparative analysis between *AIP*mut vs *AIP*neg somatotropinomas

	*AIP*mut vs *AIP*neg somatotropinomas			
	*AIP*mut	*AIP*neg		
	n = 136	n = 650	*P*	Overall somatotropinomas n = 786
Cohort type based on family history of PitNETs				
Familial cohort	63.2	34.3	**<.001**	39.3
Sporadic cohort	36.8	65.7		60.7
Gender				
Male	61.8	51.3	**.026**	53.1
Female	38.2	48.7		46.9
Age at disease onset ≤ 18 yr	67.5	25.0	**<.001**	32.7
Age at first symptoms (yr)	18.1 ± 8.4	26.1 ± 11.8	**<.001**	24.7 ± 11.7
Age at diagnosis (yr)	23.2 ± 10.8	30.2 ± 12.2	**<.001**	28.9 ± 12.3
Delay in diagnosis (yr)	4.3 ± 6.5	4.2 ± 5.4	.371	4.2 ± 5.6
Gigantism	55.9	18.2	**<.001**	24.7
Pituitary apoplexy	8.3	2.8	**.005**	3.8
Height at diagnosis (cm)				
Males	188.8 ± 19.7	183.5 ± 14.7	.054	184.8 ± 16.2
Females	170.4 ± 11.2	168.9 ± 9.0	.392	169.3 ± 9.5
Height Z-score at diagnosis	2.7 ± 2.4	1.5 ± 1.9	**<.001**	1.8 ± 2.1
Insulin-like growth factor 1 × ULN at diagnosis	2.5 ± 3.5	2.9 ± 2.3	**<.001**	2.8 ± 2.5
	*2.7 ± 3.8*	*2.9 ± 2.3*	*.696*	*2.9 ± 2.5*
Hypopituitarism at diagnosis	46.4	49.0	.742	48.3
Number of pituitary deficiencies at diagnosis	0.89 ± 1.12	0.71 ± 0.90	.565	0.76 ± 0.97
Macroadenoma	90.0	89.2	.796	89.3
Maximum tumor diameter (mm)	23.0 ± 11.9	24.8 ± 13.6	.403	24.5 ± 13.3
Suprasellar extension	60.3	46.2	**.042**	48.7
Cavernous sinus invasion	41.9	35.7	.356	36.8
Granulation pattern				
Densely granulated	0	31.9	**<.001**	22.1
Sparsely granulated	100	68.1		77.9
Ki-67 > 3%	44.0	35.7	.519	37.9
Number of treatments	2.35 ± 1.68	2.30 ± 1.41	.821	2.31 ± 1.47
Number of surgeries	1.06 ± 0.78	1.07 ± 0.61	.606	1.07 ± 0.65
Reoperation	25.2	16.1	**.025**	17.8
Radiotherapy	38.9	28.2	**.018**	30.5
Somatostatin analogues	45.4	54.2	.073	52.4
Dopamine agonists	23.8	26.3	.572	25.8
Pegvisomant	10.8	6.8	.127	7.6
Multimodal treatment	72.4	63.7	.076	65.4
≥3 treatments	45.7	36.9	.079	38.6
	*47.7*	*36.9*	***.039***	*39.0*
Active disease at last follow-up	27.7	43.3	**.005**	39.6
Hypopituitarism at last follow-up	36.1	39.1	.752	38.3
Number of pituitary deficiencies at last follow-up	0.48 ± 0.93	0.79 ± 1.22	.288	0.71 ± 1.15
Final height (cm)	185.9 ± 18.3	177.9 ± 14.3	**<.001**	179.7 ± 15.6
Final height (cm) by gender				
Males	192.8 ± 17.6	185.2 ± 13.8	**.004**	187.1 ± 15.1
Females	174.8 ± 13.4	168.9 ± 8.7	**.017**	170.1 ± 10.0
Follow-up duration (yr)	11.4 ± 12.8	7.4 ± 8.9	**.027**	8.3 ± 10.0

Categorical data are shown as %; continuous variables are shown as mean ± standard deviation. Data for clinically presenting somatotropinomas comparison are added in italics where showing different results. Data for clinically presenting somatotropinomas comparison are added in italics where showing different results. *P* values in bold are those < .05 (statistically significant).

Abbreviations: *AIP*mut, *AIP* mutation-positive; *AIP*neg, *AIP* mutation-negative; PitNET, pituitary neuroendocrine tumor; ULN, upper limit of the normal; yr, years.

### Prospectively diagnosed vs clinically presenting *AIP*mut PitNETs

Genetic testing of *AIP*mut kindreds identified 187 apparently unaffected *AIP*mut carriers. A total 165 *AIP*mut carriers were disease free at both baseline screening and at last follow-up assessment (mean follow-up duration 5.9 ± 3.3 years, ranging between 1 and 11 years), while 22 subjects (11.8%) were prospectively diagnosed with a PitNET. The mean age at diagnosis of prospectively diagnosed *AIP*mut PitNET patients (30.4 ± 15.7 years) and the age at genetic testing of unaffected *AIP*mut carriers (35.9 ± 24.1 years) did not differ (*P* = .453). There was no significant difference in the gender distribution either: 49.7% prospectively diagnosed males vs 63.6% unaffected carrier males (*P* = .219).

**Figure 2.**
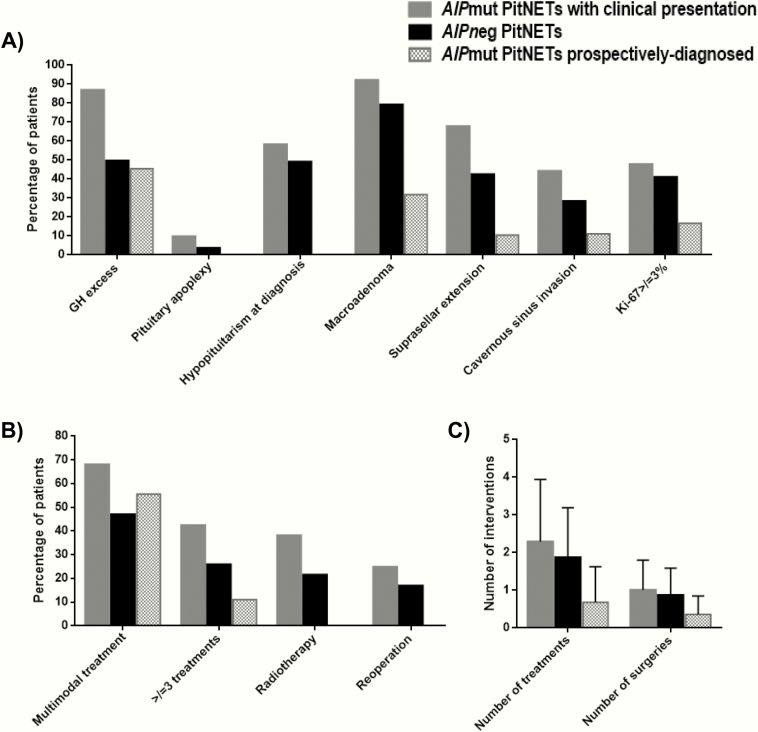
Patient characteristics (A) and treatment modalities (B,C). Clinical variables (A) and treatment characteristics (B,C) in patients with a clinically presenting PitNET, with or without *AIP* mutation (*AIP*mut and *AIP*neg), and in *AIP*mut carriers with an abnormality identified at clinical screening (prospectively diagnosed cases). (C) Data are shown as mean ± standard deviation.

Three of these prospectively diagnosed cases had normal biochemistry and contrast-enhanced pituitary MRI scan at baseline screening, but went on to develop a PitNET during the subsequent follow-up: 2 small NF-PitNETs and 1 microprolactinoma, being stable since their initial detection and none requiring intervention to date. Eight of the 22 cases (36%) had retrospectively recognized symptoms that could be attributed to pituitary disease. Prospectively diagnosed PitNETs were smaller than clinically presenting tumors (10 ± 7 vs 24 ± 13 mm; *P* < .001), and 68% vs 8% were microadenomas (*P* < .001, Table 4 and Fig. 2A). Prospectively diagnosed PitNETs were associated with lower rates of hypopituitarism at diagnosis (0 vs 58%; *P* < 0.001), suprasellar extension (11% vs 68%; *P* < .001), and cavernous sinus invasion (11% vs 44%; *P* = .010) (Table 4 and Fig. 2A). Prospectively diagnosed cases required fewer treatments (0.7 ± 1.0 vs 2.3 ± 1.7; *P* < .001) and operations (0.4 ± 0.5 vs 1.0 ± 0.8; *P* < .001), none received radiotherapy (vs 38%; *P* < .001), and had decreased rates of active disease (6% vs 28%; *P* = .039) and hypopituitarism (0 vs 41%; *P* = .003) at last follow-up (Table 4 and Fig. 2B and 2C).

**Table 4.** Comparative analysis between prospectively diagnosed vs clinically presenting *AIP*mut PitNETs

	*AIP*mut PitNETs			*AIP*mut somatotropinomas			*AIP*mut NF-PitNETs		
	Prospectively diagnosed	Clinically presenting		Prospectively diagnosed	Clinically presenting		Prospectively-diagnosed	Clinically-presenting	
	n = 22	n = 145	*P*	n = 10	n = 126	*P* value	n = 10	n = 4	*P* value
Gender									
Male	63.6	60.7	.792	70.0	61.1	.578	60.0	75.0	.597
Female	36.4	39.3		30.0	38.9		40.0	25.0	
Age at diagnosis (yr)	30.4 ± 15.7	23.5 ± 11.1	.065	32.6 ± 15.7	22.4 ± 10.0	**.022**	29.9 ± 16.3	27.0 ± 11.5	1.000
Clinical diagnosis									
Acromegaly	36.4	35.9	**<.001**						
Gigantism	9.1	51.0							
Prolactinoma	9.1	10.3							
NF-PitNET	45.4	2.8							
GH excess	45.5	86.9	**<.001**						
Hypopituitarism at diagnosis	0	58.2	**<.001**	0	54.2	**.004**	0	100	**.001**
Number of pituitary deficiencies at diagnosis	0	1.15 ± 1.19	**<.001**	0	1.04 ± 1.15	**.008**	0	2.00 ± 0	**.002**
Macroadenoma	31.8	92.1	**<.001**	60.0	92.7	**.001**	10.0	100	**.003**
Maximum tumor diameter (mm)	9.5 ± 7.2	23.8 ± 12.6	**<.001**	14.1 ± 7.6	24.5 ± 11.9	**.015**	6.4 ± 5.0	35.0	.113
Suprasellar extension	10.5	67.7	**<.001**	12.5	67.3	**.003**	11.1	100	**.011**
Cavernous sinus invasion	11.1	44.3	**.010**	14.3	45.5	.115	11.1	50.0	.197
Ki-67 > 3	16.7	47.8	.168	20.0	50.0	.227	0	50.0	.386
Number of treatments	0.68 ± 0.95	2.29 ± 1.65	**<.001**	1.20 ± 1.03	2.45 ± 1.69	**.015**	0.20 ± 6.32	1.33 ± 0.58	**.010**
Number of surgeries	0.36 ± 0.49	1.01 ± 0.79	**<.001**	0.70 ± 0.48	1.09 ± 0.79	.105	0.10 ± 0.32	1.00 ± 0	**.004**
Reoperation	0	24.8	.108	0	27.0	.112	0	0	1.000
Radiotherapy	0	38.1	**<.001**	0	42.1	**.009**	0	33.3	.057
Multimodal treatment	55.6	68.0	.443	57.1	73.4	.351	0	33.3	.248
≥ 3 treatments	11.1	42.4	.065	14.3	47.7	.085	0	0	1.000
Active disease at last follow-up	5.6	28.3	**.039**	11.1	29.3	.243	0	50.0	**.035**
Hypopituitarism at last follow-up	0	41.0	**.003**	0	40.6	.111	0	100	**.002**
Number of pituitary deficiencies at last follow-up	0	0.65 ± 1.10	**.014**	0	0.56 ± 0.97	.220	0	1.00 ± 0	**.003**
Follow-up duration (yr)	5.3 ± 4.5	12.4 ± 13.0	.067	5.5 ± 4.8	12.0 ± 13.2	.276	5.1 ± 4.7	19.5 ± 0.7	**.030**

Categorical data are shown as %; continuous variables are shown as mean ± standard deviation. *P* values in bold are those < .05 (statistically significant).

Abbreviations: *AIP*mut, *AIP* mutation-positive; NF-PitNET, non-functioning pituitary neuroendocrine tumor; yr, years.

Prospectively diagnosed somatotropinomas, NF-PitNETs and prolactinomas had significantly lower rates of hypopituitarism at diagnosis, macroadenomas, and suprasellar extension, requiring fewer treatments than those clinically presenting counterparts (Table 4). Prospectively diagnosed *AIP*mut somatotropinomas were also significantly smaller and none required radiotherapy (*P* = .009). None of the prospectively diagnosed *AIP*mut NF-PitNETs had hypopituitarism (*P* = .002) or active disease (*P* = .035) at last follow-up (Table 4). Two *AIP*mut patients had prospectively diagnosed microprolactinomas with no suprasellar extension or cavernous sinus invasion, and were eupituitary at diagnosis and at last follow-up: 1 responded well to dopamine agonist and the other is under observation (described in detail as case 5 in (14)).

### *AIP* mutations in the study population

Forty-four different germline pathogenic/likely pathogenic *AIP* mutations were identified, including 5 previously not described mutations (exon 1 deletion; c.344delT (p.L115fs*41); c.773T>G (p.L258R); c.779delA (p.K260fs*44); c.863_864del (p.F288Cfs*?)), among the 167 *AIP*mut patients (17). The most common mutation types were nonsense mutations (27%) and frameshift mutations (25%), followed by missense (18%), splice site (7%), in-frame insertions/deletions (9%), and large genomic deletions (7%), and we had 1 each of promoter, start site, and stop-loss mutations. Of 167 *AIP*mut PitNETs, 127 (76%) were due to a truncating mutation, and the most frequent *AIP* mutation was c.910C>T (p.R304*), which was detected in 57 patients.

In our study population, we identified 17 different *AIP* variants classified as benign, likely benign, or variants of uncertain significance according to the American College of Medical Genetics and Genomics and the Association for Molecular Pathology criteria (17,18). We note that one of the most common *AIP* variants identified, p.R304Q, although controversial, is currently classified as variant of uncertain significance (19), patients from these kindreds were allocated to the *AIP*neg subgroup.

### Comparative analysis of *AIP*mut vs *AIP*neg patients

Overall, patients with *AIP*mut were more frequently males (61% vs 45%; *P* < .001) than* AIP*neg patients, 8 years younger at first symptoms, and 6 years younger at diagnosis, with disease onset ≤18 years in 65% and <30 years in 87% (Table 1 and Fig. 1A). *AIP*mut PitNETs had a higher rate of pituitary apoplexy and suprasellar extension, more often required radiotherapy, and multimodal and multiple treatments than *AIP*neg ones (Table 1). Patients with *AIP*mut had lower rates of active disease at last follow-up (25% vs 35%; *P* = .041). However, as *AIP*mut had a longer follow-up, we analyzed only patients with no longer than a 10 year follow-up, and then there was no difference in the rate of active disease at last follow-up (39% vs 43%; *P* = .642).

*AIP*mut PitNETs were more often associated with GH excess, with gigantism being the predominant clinical diagnosis (Fig. 1B and (17)). *AIP*mut patients with GH excess were younger than *AIP*neg cases (Table 3). There was no difference in insulin-like growth factor 1 (IGF-1) levels at diagnosis between patients presenting clinically with *AIP*mut and *AIP*neg (*P* = .696, Table 3). All *AIP*mut somatotropinomas were sparsely granulated in contrast to 68% of the *AIP*neg ones (*P* < .001); similar ratios were seen considering only *AIP*mut and *AIP*neg giants. *AIP*mut somatotropinomas were associated with higher rates of pituitary apoplexy, suprasellar extension, radiotherapy, and reoperation, and showed trends for an increased need for multimodal therapy (*P* = .076) and ≥3 treatments (*P* = .079). The mean final height was higher in the *AIP*mut somatotropinoma subgroup both for males (193 ± 18 vs 185 ± 14 cm; *P* = .004) and females (175 ± 13 vs 169 ± 9 cm; *P* = .017) (Table 3). Patients with *AIP*mut prolactinomas had higher rates of pituitary apoplexy than *AIP*neg counterparts, which remained significantly higher when considering only clinically presenting cases (17). *AIP*mut NF-PitNETs had lower rates of macroadenomas, hypopituitarism at last follow-up, lower tumor diameter, and fewer pituitary deficiencies at diagnosis, as well as requiring fewer treatments and surgery than their nonmutated counterparts; however, when excluding the 10 prospectively diagnosed *AIP*mut NF-PitNETs patients these significant differences were lost ((17)).

## Discussion

We assessed the clinical value of genetic testing for *AIP* mutations with subsequent clinical screening of carriers in a cohort of patients with familial and young-onset PitNETs. In addition, we have compared the clinical features between *AIP*mut and *AIP*neg patients. Our key focus was on the follow-up of carriers and on prospectively diagnosed *AIP*mut patients, as the clinical and therapeutic characterization of this subgroup is lacking. The clinical screening of carrier family members of *AIP*mut probands has been recommended on the assumption that the early detection of PitNETs might be associated with more favorable outcomes (1,3,13,14); however, these predicted advantages had not been previously demonstrated in a prospective study.

In the current study, among the 187 apparently unaffected *AIP*mut carriers, 22 were identified with a prospectively diagnosed PitNET by clinical, biochemical, and imaging screening. As a group, prospectively diagnosed *AIP*mut PitNETs were mainly microadenomas, smaller, and were associated with lower rates of suprasellar extension, cavernous sinus invasion, and hypopituitarism at diagnosis, and required fewer treatments, operations, no radiotherapy, and had reduced rates of active disease and hypopituitarism at last follow-up when compared with their clinically presenting counterparts. Similar results were obtained when prospectively diagnosed *AIP*mut somatotropinomas and *AIP*mut NF-PitNETs were analyzed separately. Overall, prospectively diagnosed *AIP*mut PitNETs are significantly less invasive and associated with better outcomes than those with a clinical presentation, highlighting the benefits of *AIP* genetic testing of family members at risk and the screening of individuals carrying an *AIP* mutation (1,11,13,20).

In our series, 3 prospectively diagnosed PitNETs were not present at baseline assessment, but emerged during the follow-up (5–7 years after the initial screening), reinforcing the need for surveillance of unaffected *AIP*mut carriers (1,3,13,14). These 3 cases are currently under observation, requiring no treatment. The *AIP*mut nonfunctioning microadenomas we have identified in our study are somewhat similar to the screening-detected MEN1-related pituitary tumors described elsewhere (21). In a different study, Tichomirowa et al. identified 2 patients with PitNETs among the 21 *AIP*mut carriers screened (9.5%), both clinically silent microadenomas requiring no intervention (8). Both *AIP*mut and MEN1-related prospectively diagnosed PitNETs should be managed in accordance with current guidelines (21–27).

There are 4 key questions for clinicians managing patients with PitNET regarding AIP: (1) Which clinically presenting pituitary tumor patients should be tested for *AIP* mutations? (ii) How to manage clinically presenting *AIP*mut PitNET patients? (3) When to initiate genetic screening for family members of a proband? and (4) What should be the clinical follow-up of *AIP*mut carriers?

1. Which clinically presenting pituitary tumor patient should be tested for *AIP* mutations? We have recently showed that based on 4 simple factors (age of onset, family history, tumor type, and tumor size), the risk of carrying an *AIP* mutation can be predicted (11). As mutation status correlates with age of disease onset better than age of diagnosis (3), careful history taking is key. For example, age at onset between 19 and 30 years is an independent risk factor for patients with sporadic PitNET to carry an *AIP* mutation; however, patients in this age group without GH excess or an absence of family history have a lower risk (11). Hence, risk prediction should take several parameters into account, and for patients with fewer risk factors the age cut-off for *AIP* testing could be lower than 30 years (11,28). Our fact-finding study shows that many patients with sporadic PitNET who undergo *AIP* analysis based on age at onset ≤30 years (3–5,11) will have negative results. In our young-onset sporadic PitNET cohort, 6.8% had an *AIP* mutation, with slightly higher rates in the sporadic somatotropinoma subgroup (10.5%); this is at the level of usual risk recommendation for genetic testing, but we identified low rates in sporadic prolactinomas (1.5%) with no cases of NF-PitNETs or corticotropinomas.
2. How to manage clinically presenting *AIP*mut PitNET patients? This is an important question but is largely beyond the scope of this article. There are numerous factors which need to be taken into account due to young onset, often aggressively growing tumors, and treatment should follow current guidelines, with attention to some characteristic features, such as aggressive growth, recurrence, poor response to first-generation somatostatin analogues with, at least in some cases, better responses to second-generation somatostatin analogues (29), and the risk of apoplexy. On the other hand, some cases show slower growth or stable nonfunctioning microadenomas, as shown in our data here.
3. When to initiate genetic screening for family members of a proband? We suggest germline *AIP* mutation genetic testing be offered at the earliest opportunity to first-degree relatives including children, because the disease may manifest by the age of 4 years (30).
4. What should be the clinical follow-up of *AIP*mut carriers? Our experience, based on this cohort, suggests that careful baseline assessment of *AIP*mut carriers (including clinical examination, measurement of serum IGF-1 and prolactin, and pituitary MRI) picks up the largest number of pituitary abnormalities. As *AIP* mutation testing has only been established just over a decade ago, the age range of establishing carrier status was very wide in our cohort. However, as testing is now routinely available, we predict that a larger number of carriers will be followed starting at an early age. As the age of disease onset has an inverted U shape (Fig. 1A), the recommendation for carrier follow-up could be different for the various age groups. For *AIP*mut carriers until the age of 20 years, annual clinical assessment with measurement of IGF-1 and prolactin and baseline MRI (starting at 10 years for younger carriers) followed by 5-yearly scans could be appropriate. Follow-up between 21 and 30 years, if assessment is normal at age 20 years, probably could be relaxed. Our data also raise the possibility that adult *AIP*mut carriers with a normal baseline assessment could be followed with clinical and biochemical assessment, with further pituitary MRI only indicated in case of symptoms or biochemical abnormalities. Most clinically presenting cases show symptoms before the age of 30 years (1,3), and we are not aware of any case with a normal full assessment at age ≥30 years who later developed a PitNET. However, a cost-effectiveness analysis evaluating the economic burden of genetic testing and clinical screening programs in this setting, while weighing the benefits of early detection of *AIP*-related pituitary disease we show in this study, is currently lacking.

In the *AIP*mut and *AIP*neg comparison, *AIP*mut PitNETs presented earlier with more aggressive disease and were more difficult to treat, as seen in previous studies (3–5,10,31). Nevertheless, our data show that some *AIP*mut PitNETs will not display an aggressive phenotype (4,8,12,32). Interestingly, the inclusion of aggressive or therapy resistant pituitary disease did not increase the frequency of *AIP* mutations in a recent study (28). Moreover, in our cohort, the rate of active disease at last follow-up was 10% lower in the *AIP*mut PitNETs group, suggesting that *AIP*mut PitNETs can be satisfactorily controlled despite requiring more complex and multimodal therapeutic schemes (12,29,30,33,34). Although these data may seem paradoxical (more aggressive disease at presentation in the *AIP*mut patients, but better controlled disease at last follow-up), they could be explained by a more aggressive treatment approach in *AIP*mut cases, especially the use of radiotherapy. Another possibility is that the follow-up of *AIP*neg cases in our cohort was somewhat shorter; indeed, considering a cut-off of a maximum of 10 years of follow-up, there was no difference in rate of active disease between the 2 groups. Rostomyan et al. also reported higher rates of biochemical control at last follow-up and a trend for increased long-term controlled disease in patients with *AIP*mut pituitary gigantism in comparison to genetically negative gigantism cases (12). Thus, these data suggest that management of *AIP*mut patients can be challenging, but the disease is controllable in a significant proportion of cases.

Among *AIP*mut patients, somatotropinomas were the main PitNET subtype and gigantism the predominant clinical diagnosis, as previously shown (4, 11). IGF-1 levels at diagnosis did not differ between clinically presenting *AIP*mut and *AIP*neg somatotropinoma patients, suggesting that *AIP*mut somatotropinomas are not biochemically more active at presentation than their *AIP*neg counterparts, similar to earlier data (4). *AIP*mut patients with gigantism also showed similar IGF-1 levels in our cohort (35), although *AIP*neg giants had higher IGF-1 in another cohort (12). *AIP*mut somatotropinoma patients received radiotherapy more frequently than *AIP*neg patients, for which a nonsignificant trend had been observed previously (4). In addition, the mean final height in our cohort was higher in the *AIP*mut somatotropinoma subgroup, with both *AIP*mut males and females ending up taller than *AIP*neg counterparts, although this has not been consistently shown in other series (12). The taller final height in our *AIP*mut somatotropinoma patients is likely due to earlier onset of disease, but it may also reflect the management difficulties.

We found no differences regarding treatment and clinical outcomes in the comparative analysis of *AIP*mut vs *AIP*neg prolactinomas. Although numbers are small, this suggests that *AIP*mut prolactinomas may not be more refractory to medical therapy, in line with a previous report showing that presence of an *AIP* mutation in children or adolescents with macroprolactinomas does not influence the response to dopamine agonists (32).

*AIP*mut NF-PitNETs were smaller, had less pituitary deficiencies at diagnosis, and required fewer treatments and operations than *AIP*neg NF-PitNETs; however, these differences were lost when the 10 prospectively diagnosed cases were excluded from the analysis. In fact, clinically presenting *AIP*mut NF-PitNETs were macroadenomas, and had suprasellar extension and hypopituitarism at diagnosis/last follow-up, and half remain uncontrolled at last follow-up. Clinically presenting *AIP*mut NF-PitNETs reported previously were also noted for their aggressive behavior (4). Some of the small prospectively diagnosed *AIP*mut NF-PitNETs may represent incidentalomas similar to those often observed in the general population, although incidentalomas are more common in older subjects (2,22). Prospectively diagnosed *MEN1* mutation-positive NF-PitNETs also display an indolent behavior, do not progress to macroadenomas, and often require no intervention (21,36). Overall, our data show that not all *AIP*mut PitNETs are aggressive or difficult to manage, as some patients have slowly growing or indolent NF-PitNETs (possibly representing incidentalomas) requiring no intervention, suggesting that the spectrum of *AIP*-related pituitary disease is wider than previously suggested.

Our study has some limitations: (1) we used the onset of symptoms age cut-off ≤30 years as a criterion to guide *AIP* genetic testing in patients with young-onset sporadic PitNETs, as in previous *AIP*-related studies (3–5,11). This age cut-off relies on age of onset, which can be subjective; however, age of onset rather than age at diagnosis is suggested to be a better option to guide genetic testing as PitNETs are often diagnosed with significant delay (11); (2) our patients were recruited from several countries and thus their clinical features and outcomes may be affected by their different genetic backgrounds and/or different local clinical practices; (3) we assigned, based on current experimental, clinical and in silico data, the *AIP* variants into pathogenic/likely pathogenic, or variant of uncertain significance/likely benign/benign groups; however, these categories may change as these variants are better characterized; (4) since the apparently unaffected participants of our study were genetically and clinically screened at various ages, we cannot determine, at this point, the disease penetrance for the prospectively diagnosed cohort per age group.

## Conclusions

Genetic testing followed by clinical screening in *AIP*mut kindreds can detect clinically relevant pituitary disease, where earlier intervention results in better outcomes. While clinically presenting *AIP*mut PitNETs occur in younger patients with more advanced disease, complex treatment strategies can result in well-controlled disease. There is a wider spectrum of disease severity in *AIP*mut PitNET patients, even within the same family, than previously suspected. When considering patients for *AIP* mutation testing, key clinical factors help to predict the risk level to guide decision making.

## Abbreviations

AIP: aryl hydrocarbon receptor-interacting protein
AIPmut: AIP mutation-positive
FIPA: familial isolated pituitary adenoma
GH: growth hormone
MEN1: multiple endocrine neoplasia type 1
MRI: magnetic resonance imaging
NF: nonfunctioning
NS: not specified
PitNET: pituitary neuroendocrine tumor

## Additional Information

***Disclosure Summary:*** No potential conflict of interest.

***Data Availability:*** Restrictions apply to the availability of data generated or analyzed during this study to preserve patient confidentiality or because they were used under license. The corresponding author will on request detail the restrictions and any conditions under which access to some data may be provided.
